# Supplementary material for: Bursts in discontinuous Aeolian saltation
Source: Sci Rep. 2015 Jun 15;5:11109. doi: 10.1038/srep11109 (PMC4466891; doi:10.1038/srep11109)
Supplement: Supplementary Information [file srep11109-s1.pdf]

Supplemental Material: Bursts in discontinuous Aeolian saltation

M. V. Carneiro[1], K. R. Rasmussen[2], and H. J. Herrmann[1,3]
[1] Computational Physics, IfB, ETH Zürich, Wolfgang-Pauli-Strasse 27, 8093 Zürich, Switzerland
[2] Department of Geoscience, Aarhus University, Hoegh Guldbergsgade 2, 8000 Aarhus C, Denmark and
[3] Departamento de Física, Universidade Federal do Ceará, 60451-970 Fortaleza, Ceará, Brazil
(Dated: April 10, 2015)

I. PARTICE DYNAMICS

Trajectories and velocities of the particles are obtained iteratively by solving Newton's equations of motion through the velocity-Verlet scheme [1]. The contact between particles is described by the spring dashpot potential, which has an elastic and a dissipative contribution acting on each particle. When two particles i and j overlap (i.e. when their distance is smaller than the sum of their radii) an elastic force is applied,

$$\mathbf{F}_{el}^{(i)} = k m_i [|\mathbf{r}_{ij}| - 1/2(d_i + d_j)] \frac{\mathbf{r}_{ij}}{|\mathbf{r}_{ij}|}, \tag{1}$$

where $k = 0.5$ is a spring constant, d_i and d_j are the diameters, m_i is the mass of particle i, and $\mathbf{r}_{ij}$ is the distance vector pointing from particle i to j. A dissipation force is also applied accounting for the inelasticity of the collision,

$$\mathbf{F}_{diss}^{(i)} = -\gamma \mathbf{v}_{ij}, \tag{2}$$

where $\mathbf{v}_{ij} = \mathbf{v}_i - \mathbf{v}_j$ is the relative velocity and γ is the dissipation coefficient. The coefficient of restitution e is given by the ratio between the absolute velocities after and before the collision. For particle-wall collisions, the same forces act as, if particle i would collide with another particle of diameter d_i [2]. For simplicity, friction and rotation are neglected. For inter-particle collisions, we use $e = 0.6$. We place a reflective upper boundary sufficiently high to avoid any particle colliding against it and the lower boundary at $y = 0$, representing the deep ground, is strongly dissipative with a fixed restitution coefficient of $e_w = 0.5$. We use periodic boundary conditions in the direction of the wind to mimic an infinite system. We consider a bed of 12 particle layers to suppress the reflection of shock waves on the lower boundary due to the finite depth [3–5].

II. THE WIND PROFILE

An initial logarithmic velocity field mimics the wind profile in the absence of saltating grains in the x-direction with

$$u(y) = \frac{u_*}{\kappa} \ln \frac{y - h_0}{y_0}, \tag{3}$$

where $y_0 = D_{mean}/30$ is the roughness of the bed with D_{mean} the mean diameter of the particles, h_0 the bed height, $\kappa = 0.4$ the von Kármán constant, and u_* the wind shear velocity.

In the presence of grains, the wind strength is substantially reduced due to the momentum transferred to the grains [6, 7]. The grain stress $\tau_g(y)$ quantifies the average horizontal force per unit area f that the wind applies on the grains above y [8], i.e.,

$$\tau_g(y) = \int_y^\infty f(y')dy'. \tag{4}$$

where f is the average horizontal force per unit volume. The modified wind shear velocity $u_\tau(y)$ is the fluid stress left at the height y after the momentum transfer,

$$u_\tau(y) = u_* \sqrt{1 - \frac{\tau_g}{\rho_a u_*^2}}, \tag{5}$$

where ρ_a is the air density. To obtain the modified wind profile [7], we solve,

$$\frac{du}{dy} = \frac{u_\tau(y)}{\kappa y}, \tag{6}$$

considering $u_\tau(y)$ constant within an interval dy.

The numerical solution is achieved iteratively. The position of the bed surface is used as starting point of the integration of the wind profile. However, the particle splash and the sheet flow dynamically affect the shape of the bed surface. Consequently, we need to find h_0 for every time step. We use a criterion based on the wind values to define an approximate position for the particle surface. High density areas strongly reduce wind velocities. If the calculated velocity v_i in area y_i is below $0.1u_*$, we assume that this area contains the particle bed and the velocity is set to zero. This means that h_0 is chosen to be the point where the calculated velocity exceeds $0.1u_*$.

The wind drag is the only external force applied to the particles along the x-direction,

$$\mathbf{F}_d = -\frac{\pi D^2}{8} \rho_a C_d v_r \mathbf{v_r}, \tag{7}$$

where ρ_a is the air density and $\mathbf{v_r} = \mathbf{v} - \mathbf{u}$ is the velocity difference between particle and wind, with $v_r = |\mathbf{v_r}|$.

The drag coefficient C_d proposed by Cheng [9] is suited to model grains with irregular, natural, shapes, and is given by,

$$C_d = \left[\left(\frac{32}{Re}\right)^{2/3} + 1\right]^{3/2}, Re = \frac{\rho_a v_r D_{mean}}{\mu}, \quad (8)$$

where $\mu = 1.8702 \times 10^{-5}$ kg/(m.s) is the dynamic viscosity and Re, the Reynolds number. The vertical force acting on a particle is given by the competition between the gravity g, lift forces, and the rebouncing of particles with the ground.

III. THE TURBULENCE MODEL

The complete description of the fluid requires a definition of the turbulent flow velocity $\mathbf{u}$. Alternatively to solving the complete description of turbulence in the fluid, we keep the flow description deterministic but include a stochastic component in the trajectories. Therefore, $\mathbf{u}$ splits into the mean stream velocity $\mathbf{u}(y)$ and a stochastic part $\mathbf{u}_s$:

$$\mathbf{u} = \mathbf{u}(y) + \mathbf{u}_s. \quad (9)$$

For $\mathbf{u}_s$, several measurements [10–13] have shown a highly non-Gaussian behavior of the Lagrangian acceleration distribution of fluid particles.

The system of stochastic differential equations (SDE) for the logarithm of the dissipation rate $\chi = log(\varepsilon/\langle\varepsilon\rangle)$, with $\langle\varepsilon\rangle$ as the mean dissipation rate, reproduces the Gaussian distribution of the velocities u_t, and the highly non-Gaussian distributions for the acceleration a_t of the particles in fully developed turbulence [14],

$$d\chi = -(\chi - <\chi>)T_\chi^{-1}dt + \sqrt{2\sigma_\chi^2 T_\chi^{-1}}d\xi_1 \quad (10a)$$

$$da_t = -\left(T_L^{-1} + t_\eta^{-1} - \sigma_{a_t|\varepsilon}^{-1}\frac{d\sigma_{a_t|\varepsilon}}{dt}\right)a_t dt - T_L^{-1}t_\eta^{-1}u_t dt + \sqrt{2\sigma_u^2(T_L^{-1} + t_n^{-1})T_L^{-1}t_n^{-1}}d\xi_2 \quad (10b)$$

$$du_t = a_t dt, \quad (10c)$$

In Eq. 10, the variance of χ is approximated by $\sigma_\chi^2 = -0.354 + 0.289\log R_\lambda$ [15], where R_λ is the Reynolds number based upon the Taylor microscale defined by $R_\lambda = \sqrt{15Re}$. The mean value is given by $\langle\chi\rangle = -0.5\sigma_\chi^2$, and the relaxation time scale $T_\chi = 2\sigma_u^2/(C_0\langle\varepsilon\rangle)$.

In Eq. 10b, we use the energy containing scale $T_L = 2\sigma_u^2/C_0\varepsilon$, the energy-dissipation scale $t_n = C_0\nu^{1/2}/(2a_0\varepsilon^{1/2})$, the conditional acceleration variance $\sigma_{at|\varepsilon}^2 = a_0\varepsilon^{3/2}\nu^{1/2}$, two universal Lagrangian velocity structure constants $a_0 = 3.3$ and $C_0 = 7.0$, the kinematic viscosity ν and the velocity variance σ_u^2. The values of a_0 and C_0 are determined by demanding consistency with Kolmogorov's (1941) hypothesis [16] and fitting them to experimental data [14]. Finally there are two independent Wiener processes, i.e. Gaussian distributed random numbers, $d\xi_1$ and $d\xi_2$ with zero mean and variance dt.

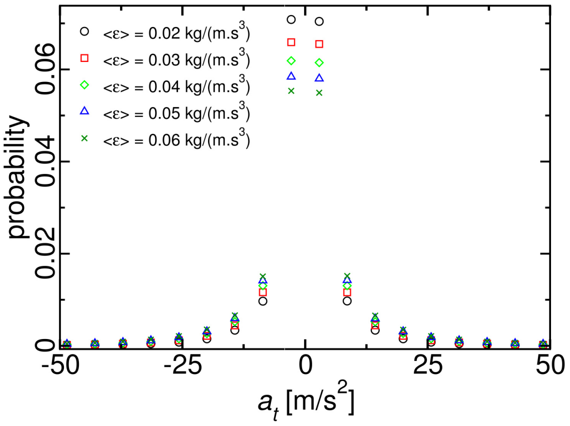

FIG. 1. (color online). Non-gaussian distributions of the random fluctuating accelerations generated by different dissipation rates $\langle\varepsilon\rangle$ with a mean a_t at 0. Smaller dissipation rates yield narrower distributions.

The noise in the stochastic differential Eq. 10 has been introduced and derived by Reynolds such as to reproduce the measured distribution of accelerations in the inertial regime. To every particle, we attach a tracer moved by fluctuating accelerations a_t by iterating Eq. 10 in time. The accelerations have probability distribution function centered at zero. Figure 1 shows the probability distribution for a_t for different dissipation rates. Smaller the dissipation rates yield narrower probability distributions. The accelerations are turned into forces and added to the net force. As in usual DEM particles do collide which is consequence of the crossing of their trajectories. As known from laboratory experiments, as for instance those of Rosato et al, the restitution coefficient and the dissipation rate is essentially constant within the range of studies [17].

This model was used also to investigate the mixing properties of two species of particles flowing through a channel [18].

IV. WIND TUNNEL SETTINGS AND WIND SHEAR VELOCITIES

Wind flow is created by a fan in the downwind end (suction) and the fan setting can be adjusted between 0 and 50 Hz with a resolution of 0.1 Hz. The span in the wind shear velocity between fluid and the impact threshold is small [19], so it is essential to precisely measure the wind shear velocity. Ten wind profiles have been recorded as function of the wind tunnel setting and the corresponding wind shear velocity which fall in the interval of approximately 0.13 m/s to approximately 0.18

m/s have been determined. From a fit to the data, shown in Fig. 2 is then possible to determine the wind shear velocity for any setting within this interval. Since measurements were taken over several months, the speed at 40 mm height has been recorded (100 Hz) in every run to ensure that no drift of the relationship take place. Although there may be a small uncertainty in the absolute value of the wind shear velocity, this procedure assures that the error in the relative difference between speed is only $0.002 - 0.003$ m/s.

Recent studies agree that in terms of dimensionless height (y/D_{mean}) the wind speed at about $y/D_{mean} = 10$ above a saltating bed is invariant for low and intermediate shear stresses [20, 21]. In our wind tunnel the height of the equilibrium boundary layer is of the order of $500D_{mean}$, and above approximately $250D_{mean}$ there is a noticeable influence from a slight wake [22]. For the wind measurements, we therefore chose an intermediate dimensionless height of approximately $200D_{mean}(\sim 40$ mm) where the wind speed depends approximate linearly on the bed shear stress.

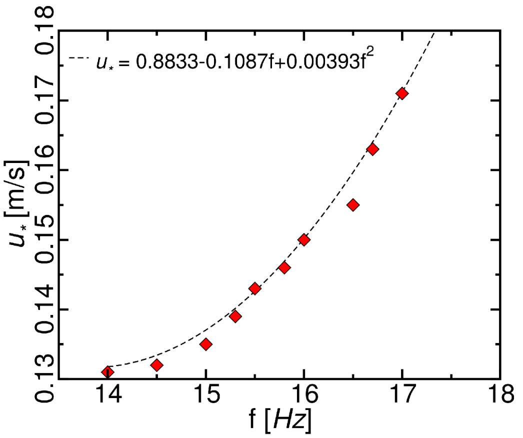

FIG. 2. (color online). Correspondence between the fan setting and the wind shear velocity measured inside the tunnel can be approximately described through the fitting function $u_* = 0.8833 - 0.1087f + 0.00393f^2$.

V. EXPERIMENT ENVIRONMENTAL CONDITIONS

Conversion of pitot tube data require information about air density which is a function of temperature, humidity and pressure which in particular may vary strongly during the same day when a depression passes. In the experiment July 2012 values were as follows: temperature 20.6 °C, pressure 1018 millibars and humidity 64%. In fall 2012 temperature was 21 °C, pressure varied from 1006-1010 millibars, and humidity was near 58%. In winter 2013 the temperature was 21.5 °C, pressure was 1013 millibars, and humidity 57%.

The environmental conditions play a very important role at the verification of the velocity threshold for sand transport. Temperature influences are important in the field, but unimportant in the laboratory where temperature is rather constant. Contrary to this, humidity may exerts some control because cohesion between particles will vary with water content (matrix potential) in the bed. In our laboratory water content in the bed increases from winter to summer because of seasonal variation in atmospheric humidity. Thus, using the findings in Ref. [23], a change from 0.170 m/s to 0.175 m/s can easily be explained as resulting from a moderate change in bed matrix potential (0.01 MPa).

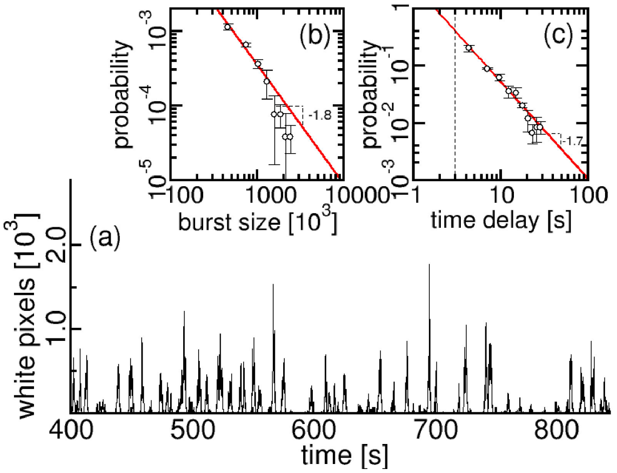

FIG. 3. (color online). (a) The time series of intermittent saltation at $\theta/\theta_c = 1.8$. The burst size distribution is fitted by a power law with an exponent -0.7. In (c), the time delay between the sand bursts follows a power law distribution with exponent -1.7.

VI. ADDITIONAL BURST STATISTICS IN INTERMITTENT SALTATION

Figure 3a and 4a show the burst activity for $\theta/\theta_c = 1.11$ and $\theta/\theta_c = 1.17$. The probability distribution for the burst sizes (in Figs. 3b and 4b) and for the time delays (in Figs. 3c and 4c) also obey power laws.

The increase of the Shields number decreases the time delay between sand bursts until saltation becomes non-intermittent. This reduces probability of larger time delays and increases of the power law exponent obtained in the distributions.

[1] Griebel, M., Knapek, S. & Zumbusch, G. *Numerical Simulation in Molecular Dynamics: Numerics, Algorithms, Parallelization, Applications* (Springer, New York, 2007).

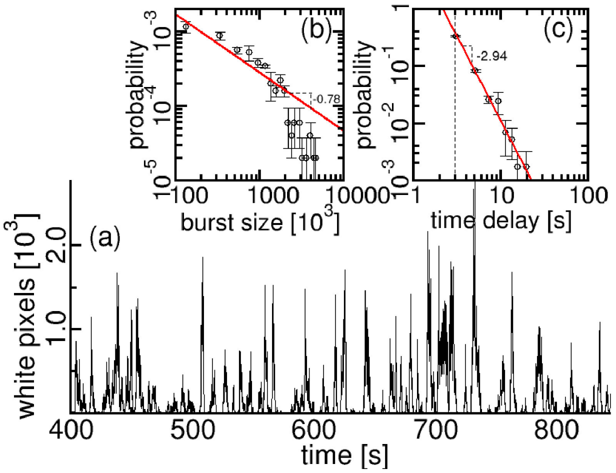

FIG. 4. (color online). (a) The time series of intermittent saltation at $\theta/\theta_c = 1.17$. The power law for the burst size distribution has an exponent -0.78. In (c), the time delay between the sand bursts obeys a power law with exponent -2.94.

[2] Herrmann, H. Simulation of granular media. *Physica A* **191**, 236–272 (1992).

[3] Rioual, F., Valance, A. & Bideau, D. Experimental study of the collision process of a grain on a two-dimensional granular bed. *Phys. Rev. E* **62**, 2450–2459 (2000).

[4] Rioual, F. Ph.D. thesis, University of Rennes 1 (2002).

[5] Rioual, F., Valance, A. & Bideau, D. Collision process of a bead on a two-dimensional bead packing: Importance of the inter-granular contacts. *Europhys. Lett.* **61**, 194–200 (2003).

[6] Anderson, R. S. & Haff, P. K. Simulation of eolian saltation. *Science* **241**, 820 – 823 (1988).

[7] Anderson, R. S. & Haff, P. K. Wind modification and bed response during saltation of sand in air. *Acta Mech.* **1**, 21–51 (1991).

[8] Ungar, J. E. & Haff, P. Steady-state saltation in air. *Sedimentology* **34(2)**, 289–299 (1987).

[9] Cheng, N. S. Simplified settling velocity formula for sediment particle. *J. Hydraul. Eng.* **123**, 149–152 (1997).

[10] Porta, A. L., Voth, G. A., Crawford, A. M., Alexander, J. & Bodenschatz, E. Fluid particle accelerations in fully developed turbulence. *Nature* **409**, 1017 (2001).

[11] Mordant, N., Crawford, A. M. & Bodenschatz, E. Experimental lagrangian acceleration probability density function measurement. *Physica D* **193**, 245 (2004).

[12] Mordant, N., Metz, P., Michel, O. & Pinton, J.-F. Measurement of lagrangian velocity in fully developed turbulence. *Phys. Rev. Lett.* **87**, 214501 (2001).

[13] Voth, G. A., Porta, A. L., Crawford, A. M., Alexander, J. & Bodenschatz, E. Measurement of particle accelerations in fully developed turbulence. *J. Fluid Mech.* **469**, 121 (2002).

[14] Reynolds, A. M. Superstatistical mechanics of tracer-particle motions in turbulence. *Phys. Rev. Lett.* **91**, 084503 (2003).

[15] Yeung, P. & Pope, S. Lagrangian statistics from direct numerical simulations of isotropic turbulence. *J. Fluid Mech.* **207**, 531 (1989).

[16] Monin, A. & Yaglom, A. *Statistical Fluid Mechanics* (MIT Press, Cambridge, MA, 1975, 1975).

[17] Rosato, A., Prinz, F., Standburg, K. & Swendsen, R. Monte carlo simulation of particulate matter segregation. *Powder Technology* **49**, 59 (1986).

[18] Burgener, T., Kadau, D. & Herrmann, H. J. Simulation of particle mixing in turbulent channel flow due to instrisic fluid velocity fluctuation. *Phys. Rev. E* **83**, 066301 (2011).

[19] Bagnold, R. A. The movement of desert sand. *Proc. R. Soc. London A* **157**, 594–620 (1936).

[20] Durán, O., Claudin, P. & Andreotti, B. On aeolian transport: Grain-scale interactions, dynamical mechanisms and scaling laws. *Aeolian Res.* **3**, 243–270 (1990).

[21] Ho, T. D., andd P. Dupont, A. V. & Moctar, A. O. E. Aeolian sand transport: Length and height distributions of saltation. *Aeolian Res.* **12**, 65–74 (2014).

[22] Coles, D. E. The law of the wake in the turbulent boundary layer. *J. Fluid Mech.* **1**, 191–226 (1956).

[23] McKenna-Neuman, C. Effects of temperature and humidity upon the entrainment of sedimentary particles by wind. *Bound.-Layer Meteor* **108**, 61–89 (2003).
